# The Mortality of Appendicitis

**Published:** 1904-01-23

**Authors:** 


### THE MORTALITY OF APPENDICITIS.

Dr. F. S. Dennis,[1] in a paper recently read before the Medical Society of the County of Kings, discussed the possibility of still further reducing the mortality of operation for the relief of appendicitis. Certain complications it is obvious are beyond the control of the surgeon, for example, crural thrombosis, intestinal obstruction, acetonuria, embolism, shock of operation, and intercurrent affections. However, to reduce the mortality by a fraction would be a distinct gain, and Dr. Dennis believes that the accumulation of experience during the last few years with regard to improved methods of technique will have such a result. There is, as he points out, some variation of opinion among the best surgeons as to the necessity of immediate operation in cases of catarrhal appendicitis. Some surgeons advocate immediate operation in all cases; others, equally distinguished, prefer to delay in the hope that the attack may quiet down, and the appendix be removed when inflammation is absent. The fact is, each individual case must be judged on its own merits. Such factors as environment, the presence of a skilled surgeon, etc., enter into the question. The general rule which Dr. Dennis recommends is to resort to immediate operation in any case which after

36 hours has not improved in any way, and in support of this position he quotes Fowler's statistics, which show that the operation mortality increases proportionately with the duration of acute symptoms previous to surgical interference. In those cases operated on within 48 hours the mortality was 17 per cent., while of those patients who were operated on in the ninth or tenth day 67 per cent. died. If, after waiting 36 hours, there is distinct improvement, it is well to wait in the hope that acute symptoms will subside, so that the appendix may be removed at a quiescent stage when the danger in skilled hands is practically nothing.

[1] Medical News, Jan. 9, 1904.

